# Paediatric urachal rhabdomyosarcoma: the role of radiotherapy about a case report and review

**DOI:** 10.3332/ecancer.2025.1852

**Published:** 2025-02-18

**Authors:** Salem Ouaddane Alami, Fatima-Zahra Abdelli, Samia Khalfi, Zenab Alami, Touria Bouhafa

**Affiliations:** Radiotherapy Department, Oncology Hospital, Hassan Ii University Hospital, Fes 30050, Morocco

**Keywords:** paediatric-oncology, radiotherapy, rhabdomyosarcoma (RMS), Urachus

## Abstract

**Introduction:**

This report discusses the case of a 10-year-old girl diagnosed with rhabdomyosarcoma (RMS) of the urachus, a rare form of soft tissue cancer in paediatric oncology. RMS, representing 3%–4% of paediatric cancers, arises from primitive muscle cells and requires a multidisciplinary treatment approach. The goal of this case is to enhance understanding of radiotherapy's role in treating RMS in children, particularly in rare sites like the urachus.

**Case:**

The patient, with no significant medical history, presented with right lower abdominal pain and was found to have a large abdominal mass. Imaging revealed a massive necrotic tumour and pulmonary metastases. The tumour was initially deemed unresectable, and a biopsy confirmed RMS. Chemotherapy was initiated using the RMS 2005 protocol, which resulted in a 70% tumour reduction. Surgical resection was then performed, and the patient received radiotherapy targeting both the primary tumour and metastases. The treatment showed no significant side effects and follow-up for a year showed no signs of recurrence.

**Discussion:**

RMS is a paediatric malignancy with poor survival rates in high-risk and recurrent cases. The urachal origin of RMS is extremely rare, with few cases reported in the literature. Management includes chemotherapy, surgery and radiotherapy. While no known tumour markers exist, associations with genetic conditions like neurofibromatosis and Li-Fraumeni syndrome have been observed. Treatment aims to cure the disease while minimizing morbidity, with surgery typically preceded by chemotherapy to reduce tumour size.

**Conclusion:**

While RMS is the most common soft tissue tumour in children, urachal RMS remains rare. Treatment involves surgery and radiotherapy, but further research is needed to establish standardized treatment protocols for such tumours.

## Introduction

This report delves into the clinical case of a 10-year-old girl diagnosed with rhabdomyosarcoma (RMS) of the urachus, a rare manifestation of soft tissue cancer that poses a particular challenge in the field of paediatric oncology. RMS, which represents about 3%–4% of all paediatric cancers [1], is characterized by the proliferation of primitive muscle cells, and it requires a complex and multidisciplinary approach to management. Through this case, we aim to deepen the understanding of radiotherapy in the context of rhabdomyosarcoma in children, thereby contributing to the enhancement of knowledge and improvement of treatment strategies for this rare localization.

## Case

It is about a 10-year-old girl with no notable medical history, admitted for further management of an alveolar-type rhabdomyosarcoma of the urachus, classed high risk, which started approximately 10 months prior to admission with right lower quadrant abdominal pain, prompting her parents to consult a paediatrician.

On initial clinical examination by paediatricians, a large abdominopelvic mass was found, occupying the entire abdomen, measuring 14 cm in its longest axis and 8 cm in its shortest axis, with no precise determination of its origin. The abdominal circumference was 54 cm, with the presence of a right inguinal lymph node measuring approximately 1 cm.

**Figure 1.**
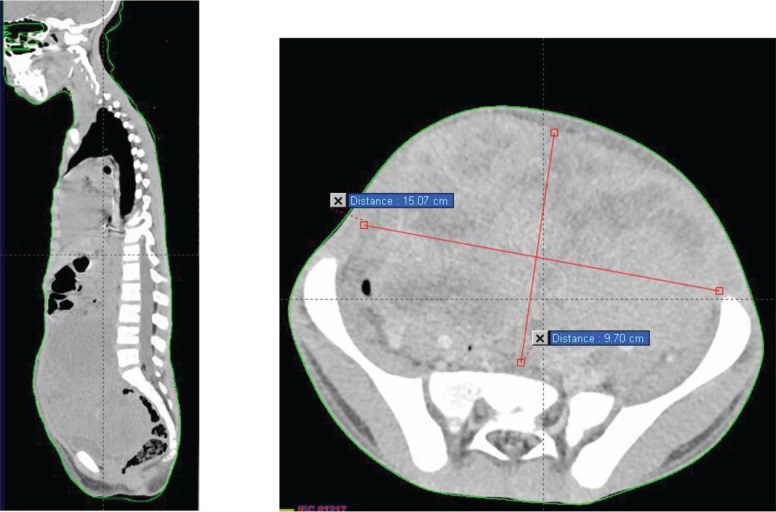
Chest–abdomen–pelvis prechemo CT scan showing a voluminous abdominal–pelvic compressive massively necrotic vascularized tumour process, responsible for compression of the adjacent digestive structures laterally and inferiorly.

A thoraco-abdominopelvic CT scan (Figure 1) revealed a large mass and pulmonary macronodules involving both lung fields, more prominently on the right side. The two largest masses measured 43 × 25 mm in the right lower lobe and 38 × 30 mm in the right upper lobe. Additionally, there were mediastinal, pre, subcarinal and pre-tracheal lymph nodes, massively necrotic, with the two largest measuring 42 × 32 mm at the right hilum and 46 × 25 mm at the subcarina. These lymph node masses exhibited similar enhancements to the previously described pulmonary masses and macronodules.

Moving to the abdominal–pelvic region, a large, massively necrotic abdominopelvic mass was observed, occupying almost the entire abdomen and pelvis, lateralized to the right, polylobed and peripherally vascularized, measuring roughly 136 × 93 × 170 mm. This mass compressed all adjacent digestive structures laterally and inferiorly, causing significant bladder displacement towards the left side. It also compressed the uterus posteriorly, as well as the rectum and rectosigmoid region.

Furthermore, compression of the right ureter with dilation of the upstream collecting system was noted, with the right renal pelvis measuring 15 mm in transverse diameter and a parenchymal index measuring 9 mm in diameter on the right. External and common iliac lymph nodes were also observed, more pronounced on the right side, the largest measuring 13 mm in diameter.

The tumour was considered unresectable, and therefore, it was decided to perform a surgical biopsy. The histopathological and immunohistochemical examination revealed an aspect of an NOS rhabdomyosarcoma.

**Figure 2.**
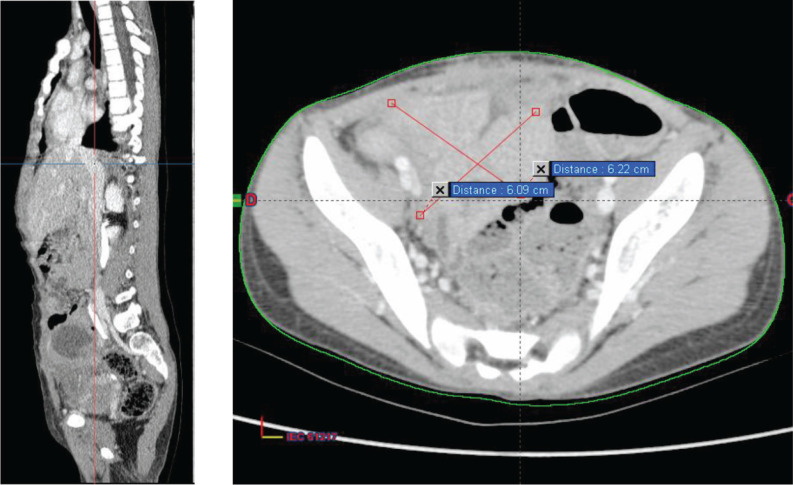
Chest–abdomen–pelvis postchemo and preoperative CT scan showed regression of the abdominal–pelvic tumour process estimated at 70%.

The decision made during the multidisciplinary team meeting was to proceed with the RMS 2005 protocol. Chemotherapy based on the IVA regimen was initiated 15 days after her admission. The patient received four cycles with good clinical tolerance, showing no mucositis, proctitis, diarrhoea, or signs of infection. Clinical examination revealed a reduction in tumour size from 10 to 7 cm, prompting the performance of a second thoraco-abdominopelvic CT scan (Figure 2) that shows a significant regression of the abdominopelvic tumour process, as well as pulmonary and nodal metastases, estimated to be over 70%.

Then, it was decided to add 2 additional cycles of chemotherapy at 21-day intervals (CEV+IVE) and to reassess with a CT scan for surgical planning. After completion of chemotherapy, a third thoraco-abdominopelvic CT scan showed that the tumour had become resectable.

**Figure 3.**
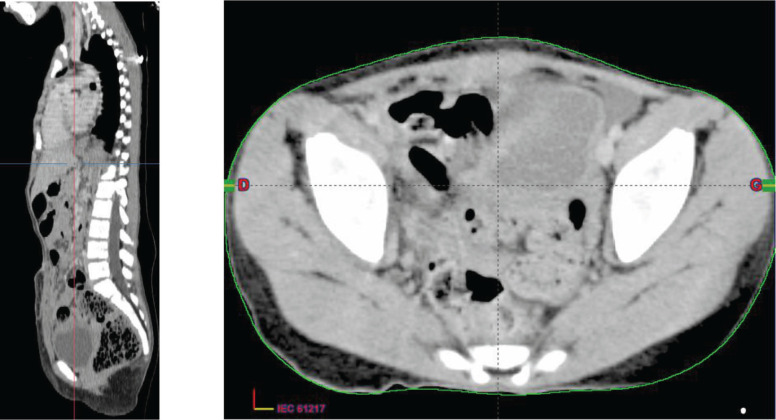
Chest–abdomen–pelvis postoperative CT scan showed the absence of any residual tumour.

Then, a surgical procedure was conducted to remove a mass measuring 6 cm in its largest dimension originating from the urachus (Figure 3).

**Figure 4.**
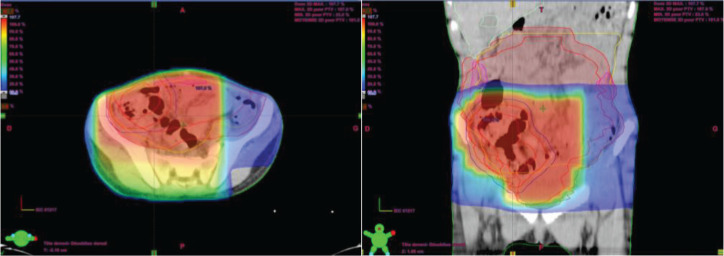
Planification and dosimetry of the tumour bed.

**Figure 5.**
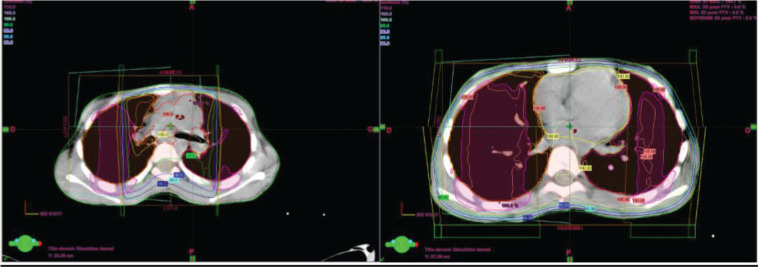
Pulmonary and mediastinal planification and dosimetry.

Two weeks after surgery, the child was referred to our radiotherapy department. The approach was to proceed with three-dimensional conformal radiotherapy (3DCRT) targeting the primary tumour volume along with the bilateral internal and iliac areas, at a dose of 41.4 Gy delivered in 23 fractions at 1.8 Gy per fraction (Figure 4). In addition, sequential 3DCRT irradiation of the pulmonary and mediastinal metastases was planned at a dose of 15 Gy, administered in 10 fractions at 1.5 Gy per fraction (Figure 5).

No side effect was observed with radiation therapy, notably neither diarrhoea nor vomiting or other complications.

The follow-up every 3 months for 1 year was marked by an improvement in the general condition of the girl without any clinical or radiological signs of recidivism.

## Discussion

RMS is a paediatric soft tissue malignancy with poor survival rates for high-risk and recurrent diseases and has the potential for significant morbidity associated with treatment [2]. It accounts for 4.5% of all cases of childhood cancer [3]. This is the third most common extracranial solid tumour of childhood after Wilms’ tumour and neuroblastoma. The median age of presentation is 6 years; however, this disease follows a bimodal distribution with peak incidences between 2 and 6 years and again between 10 and 18 years of age [4].

Although the prognosis of RMS has improved over the past 30 years due to multimodal therapies [5], the urachal origin of RMS is still extremely rare and insufficiently identified.

Few single cases were reported in the literature with only one paediatric series, including eight cases of urachal RMS in children 6]. Details about the only 13 cases of urachal RMS reported in the English literature are provided in Table 1.

While there are no known tumour markers or distinct known causes, some studies have found an association between RMS and certain environmental exposures such as prenatal X-rays and parental recreational drug use [7]. It can also be seen with increased frequency in those with Neurofibromatosis type 1, Li-Fraumeni, Beckwidth Widemann, DICER-1 syndrome, Costello syndrome and Noonan syndrome [7, 8].

The preperitoneal location permits significant asymptomatic spread within the Retzius space. This silent progression leads to a delayed diagnosis. Symptoms manifest only when the tumour reaches a large size, causing compression of adjacent organs. Urachal tumours are discovered as a hypogastric palpable mass without haematuria, except adenocarcinomas [9]. The size of the mass can make it challenging to identify its primary origin. Management of RMS should aim for a cure with minimal morbidity. Block radical cystectomy is no longer the primary treatment. Instead, the most appropriate surgical approach is considered to be total mass resection while preserving quality of life. Given that urachal RMS is often extensive, surgery is typically preceded by chemotherapy to reduce the tumour size and facilitate a less aggressive resection. Surgery and radiotherapy are the cornerstones of local treatment for RMS. Their primary goal is to cure patients while ensuring minimal long-term sequelae.

## Conclusion

Although rhabdomyosarcoma is the most common soft tissue tumour in children, tumours originating from the urachus are extremely rare. Treatment primarily involves surgery, with radiotherapy also playing a role in managing RMS. Additional research is needed to standardize the management protocol for these tumours.

## Conflicts of interest

There are no conflicts of interest.

## Funding

This article was funded by ecancer.

## Figures and Tables

**Table 1.** Clinical and anatomic pathology characteristics of urachal RMS in literature.

	Gender/age at diagnosis (years)	Presentation/general status	Urinary signs	Tumour size (mm)	Histology	IRS group	External RT dose (fields)
1) Ransom *et al* [10]	F/(0,34)	Abdominal mass/good	No	115	NOS	IV	-
2) Cheikhlared *et al* [6]	F/(5)	n/a	No	50	NOS	IV	-
3) Cheikhlared *et al* [6]	F/(3,3)	Peritoneal rupture/poor	No	100	Alveolar	IV	40 Gy (lumboaortic) 35 Gy (left iliac)
4) Cheikhlared *et al* [6]	F/(2,6)	Mass/poor	Dysuria	150	Embryonal	III	y (PTV)
5) Cheikhlared *et al* [6]	F/(4,2)	Mass/poor	Dysuria	100	Embryonal	IV	36 Gy (PTV) 48 Gy (urachus)
6) Cheikhlared *et al* [6]	M/(5,3)	Mass/poor	No	106	Embryonal	IV	36 Gy (PTV) 40 Gy (urachus)
7) Cheikhlared *et al* [6]	M/(2,5)	Mass/poor	Obstructive renal insufficiency	210	Embryonal	IV	30 Gy (abdomen in toto)
8) Cheikhlared *et al* [6]	M/(4,5)	Pain/good	No	70	Embryonal	III	-
9) Cheikhlared *et al* [6]	M/(6)	Pain/good	No	140	Embryonal	IV	30 Gy (abdomen in toto)
10) Karray [11]	M/(5)	No	Secondary enuresis	85	Embryonal		-
9 (Our case)	F/(10)	Pain/good	No	170	NOS	IV	41,4 Gy (tumour bed + EI+II) 15Gy (pulmonary and mediastinal metastasis)

NOS: not otherwise specified

II: internal iliac

EI: external iliac
